# Laryngeal histoplasmosis: report of the first case in Colombia

**Published:** 2015-12-30

**Authors:** Luz Angela Castro Alegria

**Affiliations:** Associate Professor. Bacteriology and Clinical Laboratory. Facultad de Salud, Universidad del Valle. Cali, Colombia

Dear Editor,

With regard to the article "*Laryngeal histoplasmosis: report of the first case in Colombia*" published by Moriones *et al.* *Colomb Med (Cali). 2014; 45(4):186-9* 1 *,* we would like to provide the following comments:

Histoplasmosis is a common systemic mycosis in Colombia, and because no reporting requirements exist, a national survey was designed to understand aspects of the disease that could be collected from diagnosed cases to provide useful information. A total of 434 patient surveys from 20 departments were analyzed through 2008; the majority of the reports were from Antioquia, followed by del Valle and Cundinamarca. Thus, the results from these surveys did not indicate that histoplasmosis is more commonly diagnosed in these departments, as the authors had expressed, but rather that these were the most informative departments 2, as this was a voluntary report. 

Histoplasmosis is caused by *Histoplasma capsulatum *dimorphic fungi, which present two types of pathogens to human beings:* H. capsulatum* var. *capsulatum *and *H. capsulatum* var.* duboisii* ^2^ *. *These pathogens are not acquired through the inhalation of mycelium, as explained in the article; the infecting structures correspond to hyphae fragments and principally microconidia (2-4 (m) that reach the pulmonary bronchioles and alveoli 3. It is important to clarify that the concept of mycelium refers to the mass of hyphae, and for this reason, it is not correct to suggest that this structure is responsible for infection. The microconidia are subsequently transformed into yeast, and due to the high phagocytic activity of the alveolar macrophages, they are phagocytized. They then multiply within these macrophages, sometimes leading to the destruction of the phagocyte and the infection of new cells 4. The macrophage, now carrying the microorganism, is responsible for spreading the blastoconidia to mononuclear phagocyte-rich organs. However, this does not imply that the infected macrophages are stimulated to multiply after infection, as is presented in the article. 

In paragraph six of the discussion, the authors state that in the Periodic Acid-Schiff (PAS) stains and in the Gomori-Grocott and Gridley's methenamine silver stains, the characteristic structures of *H. capsulatum* consist of hyphae within the macrophages. This statement is based on a reference and is imprecise, as it is widely known that the fungus within the macrophages is presented as blastoconidia, which are characterized as being oval shaped, 2-4 (m, and unigeminate 2. However, the presence of hyphae accompanied by blastoconidia has been occasionally reported and is considered a rare finding 5. 

Paragraph seven of the discussion provides notions surrounding the sensitivity and specificity of the test applied to different specimens and types of patients. However, it is generally concluded that the antigenic detection tests "are useful but only if they are positive because sensitivity is poor". This conclusion contradicts the generally accepted concept of the high sensitivity and specificity of the tests that detect antigens in the diagnosis of disseminated histoplasmosis 2 ^. ^This is a useful assay for not only the rapid diagnosis of this clinical form but also evaluating the response to treatment. Because the levels of detectable antigen are related to the severity of the clinical presentation, the sensitivity of this method is greater in patients with an immune system that is weakened by the high concentration of the microorganism. Detection of this microorganism in urine and blood could produce similar sensitivity values in the disseminated form of the disease 6. 

With respect to the role of polymerase chain reaction (PCR) for the diagnosis of histoplasmosis, it is not uncertain, as is suggested in paragraph ten of the discussion. Molecular tests have been developed to detect the DNA of *H. capsulatum* utilizing PCR techniques, with some having a high sensitivity and specificity 7. Recently, the Medellin Biological Research Corporation (Corporación para Investigaciones Biológicas en Medellín) in Colombia has validated and implemented a PCR test used for the identification of *H. capsulatum* in different clinical samples, which has a sensitivity of 100% and a specificity of between 92.4% and 95.2% 8.

## Editor Note:

Authors did not responding to this letter to editor
